# Asthma among WTC Children: Registry Yields First Child Health Report

**DOI:** 10.1289/ehp.116-a440a

**Published:** 2008-10

**Authors:** Cynthia Washam

The World Trade Center Health Registry (WTCHR), comprising persons most likely to have been heavily exposed to traumatic events and air pollution related to the World Trade Center attacks of 11 September 2001, includes 3,184 children under age 18 years who were living or attending school in lower Manhattan at the time, who were otherwise near the World Trade Center that morning, or who assisted in recovery efforts. In the first report of children enrolled in this registry, researchers observe that preschoolers exposed to smoke and dust from the collapsing towers had asthma rates twice the national average following the 9/11 attack, whereas asthma rates in exposed older children remained about average **[*EHP* 116:1383–1390; Thomas et al.]**.

Children in certain ethnic groups also experienced disproportionate asthma rates, although the reasons for this are unclear. Data were collected in 2003 and 2004 by telephone interviews with parents of younger children or the children themselves if they had turned 18 since the attacks. More than half the children reported having respiratory symptoms after the attacks, including cough and sinus problems. Nearly 6% of all children reported having asthma diagnosed after 9/11. At the time of the interviews, 16% of children then aged 2–4 years had been diagnosed with asthma, more than twice the average of 7% for children that age in the Northeast. Asthma rates in older subjects, however, were just slightly higher than the Northeast rate.

Childhood asthma normally develops in a child’s first five years of life, often after exposure to an environmental irritant. Smoke and dust from the collapsing towers might have acted as such an early trigger in susceptible preschoolers. The researchers speculate that older youngsters could have had fewer new diagnoses because most susceptible children had been diagnosed before 9/11.

The researchers noted racial disparities in asthma rates. Black and Hispanic children in the WTCHR were twice as likely to be diagnosed with asthma as whites or Asians, both before and after the attacks. Reasons for the racial disparities are unclear, although prior studies on ethnic disparities in asthma suggest that both genetics and environment may play a role in etiology of the disease. Children of all ages and ethnicities were more likely to develop asthma if they were caught in the cloud of cement dust created by the collapsing towers, as pulverized cement dust is known to irritate mucous membranes.

The WTCHR data have several limitations, including lack of information on how long after 9/11 symptoms appeared and the presence of co-factors for asthma. Despite these limitations, findings from the WTCHR, which constitutes the largest collection of post-disaster data of children, could have broad impact, given that tens of thousands of New York City children may have been exposed to smoke and dust on 9/11. Researchers also expect their data to improve understanding of risks to children exposed to other polluting disasters, such as the California wildfires.

## Figures and Tables

**Figure:**
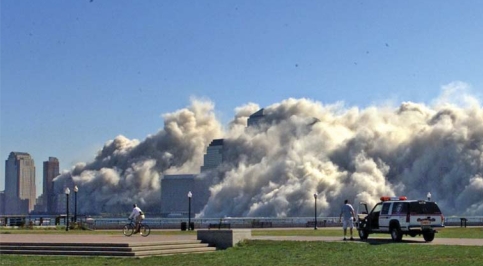
Collapse of the World Trade Center towers, 11 September 2001

